# The effects on pain, physical function, and quality of life of quadriceps strengthening exercises combined with Baduanjin qigong in older adults with knee osteoarthritis: a quasi-experimental study

**DOI:** 10.1186/s12891-021-04179-8

**Published:** 2021-03-29

**Authors:** Fenglan Wang, Xiaoli Zhang, Xiao Tong, Min Zhang, Fengmei Xing, Kun Yang, Nana Jiao, Zhiguang Duan

**Affiliations:** 1 School of Nursing, Shanxi Medical University, 56 Xinjian Road, Yingze District, Taiyuan, 030001 China; 2 College of Nursing and Rehabilitation, North China University of Science and Technology, 21 Bohai Road, Caofeidian District, Tangshan, 063210 China; 3 Department of Joint Surgery, The Second Hospital of Tangshan, 21 Jianshe Road, Lubei District, Tangshan, 063000 China

**Keywords:** Knee osteoarthritis, Baduanjin qigong, Quadriceps strengthening exercises

## Abstract

**Background:**

Exercise is recommended as a principal treatment for individuals with knee osteoarthritis (KOA). However, the best choice for an optimal exercise program able to promote long-term compliance in KOA patients is not clear. This study aims to compare the effect of combined exercise (CE: quadriceps strengthening exercises (QSE) and Baduanjin qigong training (BDJ)) versus QSE alone and BDJ alone on older adults with KOA.

**Methods:**

A three-arm, quasi-experimental trial with repeated measurements was used. As a cluster randomized trial, participants from three community centers were assigned respectively to QSE group, BDJ group and CE group. We assessed pain intensity, physical function, self-efficacy, and health-related quality-of-life (HRQoL) using standardized instruments at baseline, 3 months and 6 months follow-up.

**Results:**

One hundred and twenty-eight participants with KOA aged over 60 completed the study. Over the 6 months, there were significant group interaction effects on pain intensity (F = 28.888, *P* < 0.001), physical function (F = 26.646, *P* < 0.001), and self-efficacy (F = 22.359, *P* < 0.001), and, based on a short form-12 item health survey questionnaire (SF-12), physical component summary (F = 7.470, *P* < 0.001), and mental component summary (F = 10.207, *P* < 0.001). Overall, the CE group exhibited significantly greater improvement in all outcomes when compared to the QSE group and the BDJ group.

**Conclusions:**

CE treatment is more effective than QSE and BDJ in pain relief, increasing physical function, improving self-efficacy, and raising quality-of-life in community-dwelling KOA older adults. Moreover, it promotes long-term compliance in KOA community patients.

**Trial registration:**

Chinese Clinical Trails Registry number ChiCTR2000033387 (retrospectively registered). Registered 30 May 2020.

**Supplementary Information:**

The online version contains supplementary material available at 10.1186/s12891-021-04179-8.

## Background

Osteoarthritis (OA), the third highest risk factor for disability in the elderly, can lead to pain, loss of function, and reduced quality-of-life (QoL) [1]. As the global population ages, the number of elderly with OA is increasing in both low- and high-income countries [2]. Globally, knee osteoarthritis (KOA) accounts for over 80% of OA patients [3]. In the USA, KOA affects approximately 37% of American adults aged over 60 years [4]. In China, years lived with disability (YLDs) for KOA per 100,000 population was 968 in 2012, with 60% of YLDs contributed by individuals aged over 60 years [5].

As there is no way to cure KOA [1], treatment of KOA mainly focuses on pain reduction and improving physical function and quality-of-life [6, 7]. Exercise therapy is perhaps the most efficient non-pharmacologic treatment for KOA due to its low-cost, high safety, and facile operation [7, 8]. The KOA clinical guidelines issued by the American College of Rheumatology, states exercise interventions, including aerobic, resistance, and aquatic exercise are highly recommended [9].

Among recommended exercise programs, quadriceps strengthening exercise (QSE) has been used widely in KOA patients. Previous studies indicate QSE has better short-term effects on the relief of joint pain and stiffness and in improving QoL [7, 10, 11]. However, long-term adherence to exercise regimens is a barrier to the widespread deployment of QSE [11, 12]. For example, 44.2% of KOA patients withdrew from exercise intervention due to time, complexity of the intervention program, or other reasons [10]. The type of exercise preferred by patients and the way in which exercise is delivered may affect adherence [12]. Thus, the optimal way to promote long-term exercise adherence in KOA patients must be identified.

Mind-body exercises including Tai Chi, Yoga, Baduanjin, and Qigong have become increasing popular in pain management [13]. When managing KOA, Tai Chi is recommended as an appropriate exercise intervention [9]. Baduanjin, a well-known form of traditional Chinese Qigong, is similar to Tai Chi but requires fewer movements and a shorter time [14]. Studies indicate that Baduanjin has physiological and psychological effects in various disease states, reducing the symptoms of morning stiffness, spinal pain, and fatigue for patients with ankylosing spondylitis [15], alleviated musculoskeletal pain in older people with chronic low back and neck pain [16, 17], and improving mental health in many participants [18]. A recent review assessing the safety and efficacy of Baduanjin for KOA found only three randomized controlled trials [14]. It showed that Baduanjin can be a simple and appropriate low-intensity aerobic exercise for elderly KOA patients, but the effect of Baduanjin on improving OA symptoms requires additional investigation.

A review indicates that traditional exercise training focuses on improving muscles strength rather than modulating balance deficits and stress management, which are key factors related to the mobility of KOA patients [19]. Baduanjin, as a mind-body exercise modality, when combined with QSE, may enhance muscles strength, balance, mental health, and stress management.

The Ottawa panel clinical practice guidelines for managing KOA point out that the combination of aerobic exercise and strengthening exercises can significantly improve pain relief and physical function [20]. However, relatively little robust research has been undertaken on the combination of Baduanjin with QSE in KOA patients. This study aims to explore the effect of combining Baduanjin with QSE on older adults with KOA. Our hypothesis is that a combination exercise program could relieve pain and improve physical function, consequently improving self-efficacy and QoL, while also promoting long-term compliance to the exercise regime. Should the outcome of testing this hypothesis be valid, our study would suggest to clinical staff and rehabilitation therapists that when they write exercise prescriptions for KOA patients, rather than focusing solely on exercise programs to improve muscle strength, they should pay equal attention to combined exercise programs producing synergistic effects.

## Methods

### Study design

This was a quasi-experimental, assessor-blinded trial comprising three parallel groups with repeated measurements. The study was conducted in three community health centers in Tangshan city, China, labelled community A, community B, and community C. Post-test data were collected at 3 months and 6 months after baseline. Prior to any interventions, all researchers received professional training to provide QSE and Baduanjin instruction to the subjects.

### Participants

This study recruited community-dwelling elderly patients with KOA using print and social-media advertisements. After being registered in community centers by research assistants, clinicians screened volunteers using defined inclusion and exclusion criteria to determine initial study participants.

Inclusion criteria were: participants were (1) aged over 60 years, (2) clinically diagnosed with KOA, (3) had experienced knee pain on most days of the previous month, and (4) knee pain in the past week was between 3 and 7 on an 11-point numeric rating scale (NRS). Exclusion criteria were: (1) severe/uncontrolled comorbidities, such as myocardial ischemia, unstable angina pectoris, or mental illness, (2) neurological disorders affecting the lower limbs, (3) joint replacement surgery, (4) acute trauma of knee joint, (5) severely deformed lower limbs, and (6) having received intraarticular injection within the past 3 months. As participants should have received no Baduanjin qigong training or quadriceps muscle strength exercise before entering the trial, if subjects performed any kind of sports for at least 20 min twice a week, for more than 1 month regularly, they were categorized as regular exercisers, and were also excluded.

### Randomization and blinding

To avoid contamination effects among participants from the same community, we used randomization by community, randomizing the three communities by drawing lots. Nine equal-sized sheets of paper read either “QSE intervention in community A” or “QSE intervention in community B” or “QSE intervention in community C” or “BDJ intervention in community A” or “BDJ intervention in community B” or “BDJ intervention in community C” or “CE intervention in community A” or “CE intervention in community B” or “CE intervention in community C”. These sheets were packaged into groups of three and placed into three opaque envelopes. After recruitment, two envelopes were selected randomly and opened by a research assistant. Assessors and statisticians were blinded to participant allocation. They did not participate in the recruitment or intervention process.

### Sample size

The required sample size was calculated using Power Analysis and Sample Size (PASS 15) software set for repeated measures. The primary outcome was defined as change in the pain dimension of the Western Ontario and McMaster Universities Arthritis Index (WOMAC) among the three study groups at the end of three and 6 months. Assuming that the mean difference and standard deviation (SD) among the study groups was equivalent to that from a prior study on community-dwelling KOA patients [21], a minimum of 31 participants per group were required for a medium effect size at a power of 80% and a significance level of 0.05. To account for a projected dropout rate of 15%, we sought to include 50 participants per group.

#### Interventions

##### Combined exercise (CE: quadriceps strengthening exercises and Baduanjin qigong)

The CE group comprising participants who undertook QSE plus Baduanjin training program at least three times weekly over 6 months. They were asked to complete a brief exercise diary, which recorded the number of exercises per day. The combined QSE and Baduanjin training consisted of two phases: a face-to-face phase (0–6 weeks) and a follow-up phase (6 months). In the face-to-face phase, participants attended a 2 h class conducted by trained researchers twice weekly for 6 weeks. The main points of QSE and Baduanjin were explained and step-by-step instruction provided. Each class was conducted in groups of 10–15 participants. Each class included an hour for QSE and an hour for Baduanjin. Outside of these sessions, participants were encouraged to practice the exercises themselves. In the follow-up phase, participants were expected to practice QSE at home and practice Baduanjin in groups, at least three times weekly until the end of the study. Scheduled follow-up telephone calls were undertaken each week by research assistants in weeks 7 to 12, and then each month in weeks 13 to 24. Telephone calls discussed participant progress, identifying barriers, and encouraged subjects to adhere to the exercise program. Participants were also encouraged to contact researchers should they have concerns or questions.

##### Quadriceps strengthening exercises (QSE)

The QSE program was created after a literature review [22, 23], clinical practice, and consultation with experts. It has been shown to be safe, effective, and straightforward for study participants. QSE exercises undertaken are detailed in Additional file 1. The participants with KOA were asked to practice QSE 30–40 min per day for at least 3 days per week.

##### Baduanjin qigong

All Baduanjin exercises used were recommended by the Chinese Health Qigong Association in 2003, and consisted of eight postures [14]. It was recommended to practice two sets, once per day [14, 15], requiring approximately 20 min per set. Participants were asked to practice Baduanjin in a group-based form at least 3 days per week, except for those unable to leave home, who practiced at home. Participants were told that exercises should be performed within a tolerable level of pain. If discomfort was experienced while exercising, researchers would undertake a re-assessment, adjusting existing plans based on participants’ physical functioning and knee symptoms.

##### Baduanjin qigong training (BDJ)

The BDJ group were trained to use Baduanjin qigong exercising and received telephone follow-ups similar to those of the CE group. During the trial period, participants in the BDJ group did not practice quadriceps strengthening exercises or other types of qigong exercise, Tai Chi, or other similar exercises. However, their daily activities were not otherwise restricted.

##### Quadriceps strengthening exercises (QSE)

The QSE group were trained to use QSE exercises and had telephone follow-ups similar to those received by the CE group. During the trial period, participants in the QSE group did not practice Baduanjin or other types of qigong exercise, Tai Chi, or other similar exercises. Their daily activities were not otherwise restricted.

### Outcome measurement

Outcome measures were assessed at baseline, 3 months, and 6 months by the same research assistants who had previously been blinded to intervention allocation. Demographic variables were age, gender, height, weight, body mass index (BMI), disease duration, and social circumstances.

### Primary outcome measures

The primary outcomes were: pain intensity and physical function related to KOA, as measured by WOMAC [24]. The WOMAC index was designed for use in clinical trials of patients with osteoarthritis of the hip and knee. It includes 5 items on pain and 17 items on physical function, rated on a 0–4 Likert scale. Higher scores reflect greater pain, stiffness, and increased difficulty in physical function. The internal reliability of the Chinese version of WOMAC, as measured using Cronbach’s alpha, is 0.67–0.82 for its three subscales [25].

### Secondary outcome measures

Participants’ confidence in managing arthritis symptoms was measured using the 8-item version of the Arthritis Self-Efficacy Scale (ASES-8) [26]. This consists of eight items with no subscales. Responses were averaged, producing a score from 1 (very uncertain) to 10 (very certain), thus a high score indicated greater self-efficacy. The internal consistency of the Chinese ASES-8, as measured by Cronbach’s alpha, is 0.942 [27].

HRQoL was measured using a Short Form 12 questionnaire (SF-12), comprising 12 items within two domains: physical component summary (PCS) and mental component summary (MCS) [28]. Higher scores indicate better physical and mental health [29]. SF-12 has been widely used to assess HRQoL in patients with various conditions, including KOA disease, demonstrating good reliability and validity [30]. The Chinese SF-12 has good internal consistency, with a Cronbach’s α of 0.86 [29].

### Statistical analysis

Data were analyzed using SPSS version 23.0 (IBM Corporation, Armonk, NY, USA). Descriptive statistics such as means, SDs, frequencies, and percentages, were used to summarize participant demographic and clinical characteristics. For intervention effects, repeated measure analysis of variance was used to determine the significance of differences at 6 months in pain, physical function, self-efficacy, and HRQoL among the three groups. The interaction effect (group × time) assessed the change among groups in outcome variables across three time points. A Sidak statistical adjustment was used to minimize possible errors from multiple pairwise comparisons. The statistical significance level was set at 0.05.

## Results

**Fig. 1:**
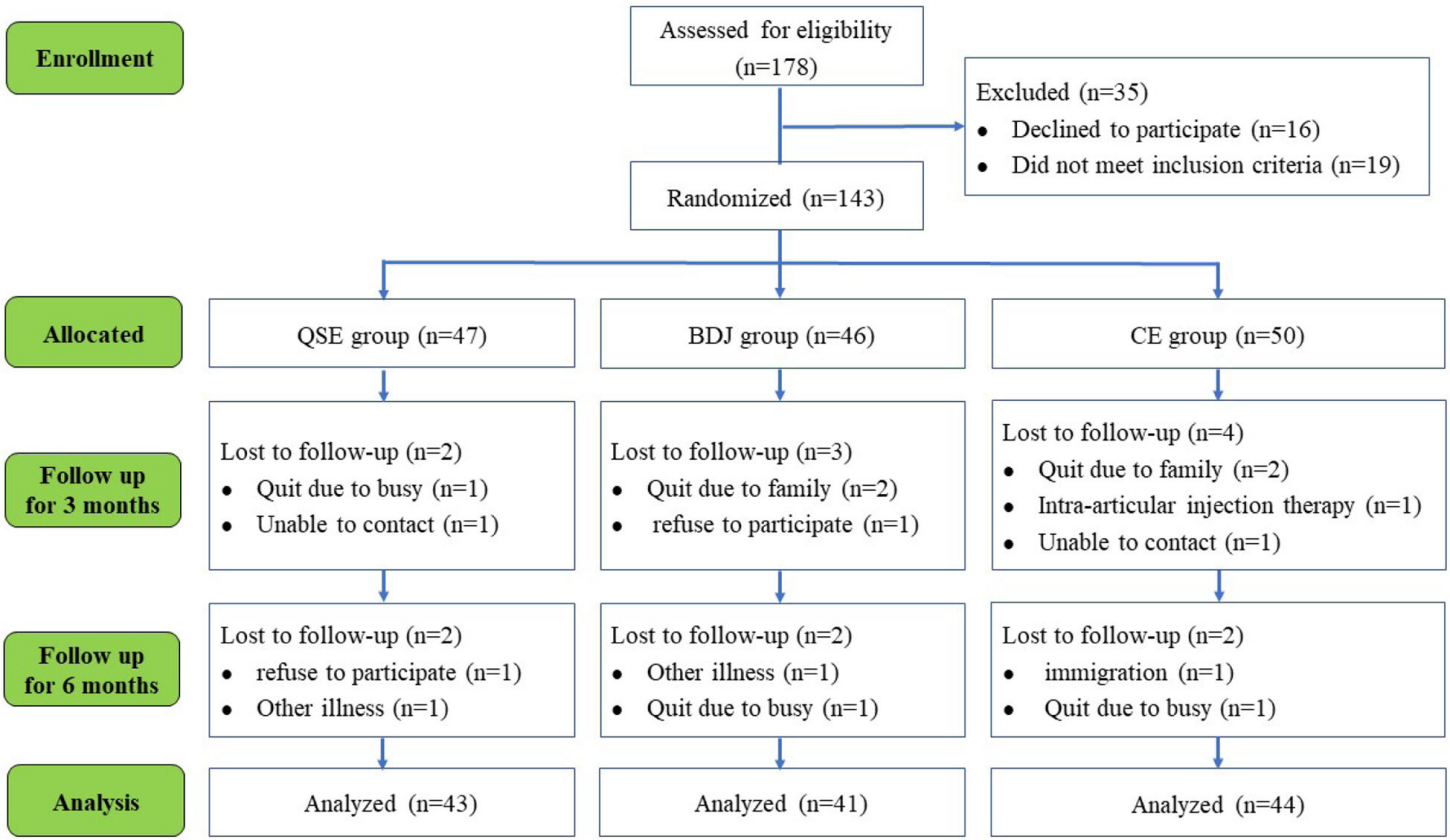
Flowchart of the study participants. QSE: quadriceps strengthening exercise; BDJ: Baduanjin qigong; CE: the combined exercise of quadriceps strengthening exercise and Baduanjin qigong

One hundred and seventy-eight eligible participants from three community centers were assessed. Thirty-five were excluded: 19 did not meet selection criteria and 16 did not wish to participate. This left 143 participants who met the study criteria and agreed to participate, with 50 in the CE group, 47 in the QSE group and 46 in the BDJ group. During the study, 15 of the 143 participants were lost to follow-up (10.5%), as shown in Fig. 1.

### Participant characteristics

Demographic characteristics of the participants are given in Table 1. The mean age was 65.34 (SD = 3.17; range 60–72). 84.4% of subjects were female and 15.6% were male. The mean duration of symptoms was 8.02 (SD = 3.73; range 2–16). 32.0% of participants had two affected knees. There were no statistically significant differences in demographic or clinical data among the three groups at baseline (Table 1). At 6 months, five participants (11.4%) exercised less than three times per week in the CE group, 13 subjects (30.2%) in QSE group and nine subjects (22.0%) in BDJ group. There were statistically significant differences between CE and QSE groups (χ^2^ = 4.719, *P* = 0.036). No statistically significant differences were observed between BDJ vs QSE (χ^2^ = 0.745, *P* = 0.270) and BDJ vs CE groups (χ^2^ = 1.729, *P* = 0.153).

**Table 1:** Baseline demographic and clinical characteristics among the three groups (*n* = 128)

Characteristic	Total(*n* = 128)	QSE(*n* = 43)	BDJ(*n* = 41)	CE(*n* = 44)	*χ2/F*	*P*-value
Age, Mean (SD)	65.34 ± 3.17	65.70 ± 3.50	64.74 ± 2.80	65.57 ± 3.15	1.206 ^a^	0.303
Gender (n, %)					0.961 ^b^	0.619
Male	20 (15.6)	8 (18.6)	7 (17.1)	5 (11.4)		
Female	108 (84.4)	35 (81.4)	34 (82.9)	39 (88.6)		
Marital status (n, %)					0.642 ^b^	0.725
Married	109 (85.2)	37 (86.0)	36 (87.8)	36 (81.8)		
Single/Divorce/ Widowed	19 (14.8)	6 (14.0)	5 (12.2)	8 (18.2)		
Educational level (n, %)					0.583 ^b^	0.997
Primary education or blow	27 (21.1)	9 (20.9)	8 (19.5)	10 (22.7)		
Secondary education	50 (39.1)	18 (41.9)	15 (36.6)	17 (38.6)		
Higher education	35 (27.3)	11 (25.6)	12 (29.3)	12 (27.3)		
College or above	16 (12.5)	5 (11.6)	6 (14.6)	5 (11.4)		
Monthly income RMB					0.434 ^b^	0.980
≤ ¥999	22 (17.2)	7 (16.3)	8 (19.5)	7 (15.9)		
¥1000 ~ 2000	62 (48.4)	20 (46.5)	20 (48.8)	22 (50.0)		
≥ ¥2000	44 (34.4)	16 (37.2)	13 (31.7)	15 (34.1)		
BMI, Mean (SD)	23.96 ± 2.03	24.12 ± 2.13	23.94 ± 2.02	23.83 ± 1.96	0.222 ^a^	0.801
Symptom duration, Mean (SD)	8.02 ± 3.73	8.16 ± 4.01	7.80 ± 43.47	8.07 ± 3.75	0.102 ^a^	0.903
Number of affected knees					0.798 ^b^	0.671
One	87 (68.0)	27 (62.8)	29 (70.7)	31 (70.5)		
Two	41 (32.0)	16 (37.2)	12 (29.3)	13 (29.5)		

^a^ one-way ANOVA; ^b^ Chi-square; *BMI* Body mass index, *QSE* Quadriceps strengthening exercise, *BDJ* Baduanjin qigong, *CE* The combined exercise of quadriceps strengthening exercise and Baduanjin qigong, *SD* Standard deviation; *p* < 0.05 was considered statistically significant

### Effects on WOMAC pain intensity and physical function

No statistically significant differences among the three groups were found for pain intensity or physical function at baseline and at month 3 of follow-up (see Table 2). At month 6 of follow-up, there were statistically significant differences among groups for both pain (F = 9.071, *P* < 0.001) and physical function (F = 5.440, *P* = 0.005).

**Table 2:** Comparisons the average score of pain and physical function among the three groups in different time points (*n* = 128)

Primary outcomes	BL	3rd MFU	6th MFU	Between groups (QSE vs. BDJ vs. CE)						Time × Group	
				BL		3rd MFU		6th MFU			
	(Mean ± SD)	(Mean ± SD)	(Mean ± SD)	*F*	*P*-value	*F*	*P*-value	*F*	*P*-value	*F*	*P*-value†
WOMAC-pain				0.045	0.956	2.875	0.060	9.071	< 0.001	28.888	< 0.001
QSE(*n* = 43)	6.91 ± 2.42	5.47 ± 2.20	5.53 ± 2.49								
BDJ(*n* = 41)	6.76 ± 2.42	5.71 ± 2.10	5.46 ± 2.25								
CE(*n* = 44)	6.86 ± 2.24	4.66 ± 2.08	3.68 ± 2.13								
WOMAC-physical function				−0.061	0.941	2.746	0.068	5.440	0.005	26.646	< 0.001
QSE(*n* = 43)	18.91 ± 5.35	16.74 ± 5.07	16.88 ± 6.49								
BDJ(*n* = 41)	18.76 ± 5.06	16.32 ± 5.05	16.00 ± 6.45								
CE(*n* = 44)	19.14 ± 4.77	14.41 ± 4.72	12.59 ± 6.27								

**†** The repeated measures ANOVA was used to test the time by group interaction effects across the 3 time points of the study

*WOMAC* Western Ontario and McMaster Universities osteoarthritis index, pain (0–20), physical function (0–68), *QSE* Quadriceps strengthening exercise, *BDJ* Baduanjin qigong, *CE* The combined exercise of quadriceps strengthening exercise and Baduanjin qigong, *BL* Baseline, *MFU* Month of follow-up, *SD* Standard deviation; *p* < 0.05 was considered statistically significant

Repeated measures-ANOVA was undertaken to test for time-by-group-interaction effects on pain intensity and physical function for the length of the whole study. Results are presented in Table 2. This analysis indicated significant time-by-group-interaction effects on pain (F = 28.888, *P* < 0.001) and physical function (F = 26.646, *P* < 0.001).

**Fig. 2:**
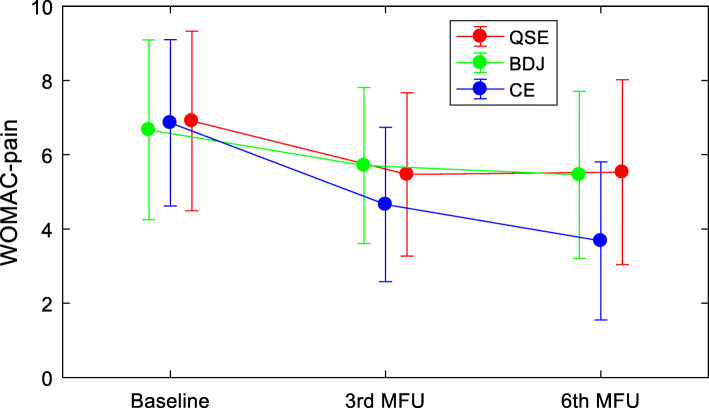
Trends of pain intensity for the three groups participants at the 3 time points of the study. WOMAC: Western Ontario and McMaster Universities osteoarthritis index; QSE: quadriceps strengthening exercise; BDJ: Baduanjin qigong; CE: the combined exercise of quadriceps strengthening exercise and Baduanjin qigong; MFU: month of follow-up

**Fig. 3:**
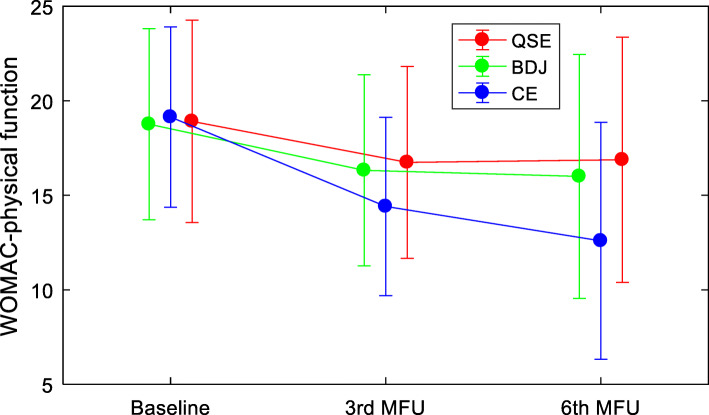
Trends of physical function for the three groups participants at the 3 time points of the study. WOMAC: Western Ontario and McMaster Universities osteoarthritis index; QSE: quadriceps strengthening exercise; BDJ: Baduanjin qigong; CE: the combined exercise of quadriceps strengthening exercise and Baduanjin qigong; MFU: month of follow-up

Figures 2 and 3 illustrate time-by-group-interaction effects and show the trends for pain and physical function, across the three study time points. Among the three groups, scores for pain and physical function at the end of follow-up were significantly reduced when compared to baseline, with the CE group having significantly larger reductions in both measures when compared to the other two groups.

Post-hoc multiple comparison of differences in pain and physical function during the study is shown in Table 3. For the CE group, there were statistically significant reductions in pain intensity and physical function over different time intervals: Baseline vs month 3 of follow-up: pain: 2.205 (CI: 1.896–2.514), physical function: 4.727 (CI: 4.194–5.260). Baseline vs month 6 of follow-up: pain: 3.182 (CI: 2.803–3.561), physical function: 6.545 (CI: 5.598–7.493). Month 3 of follow-up vs month 6 of follow-up: pain: 0.977 (CI: 0.639–1.315), physical function: 1.818 (CI: 0.943–2.694). In the other two groups, statistically significant reductions in both measurements were seen for the same time intervals, except at month 3 of follow-up vs month 6 of follow-up (QSE group: pain: -0.070 (CI: − 0.412-0.272), physical function: -0.140 (CI: − 1.025-0.746), BDJ group: pain: -0.244 (CI: − 0.106-0.594), physical function: -0.317 (CI: − 0.394-1.028)).

**Table 3:** SIDAK multiple pairwise comparisons of average score of pain and physical function for the three groups at different time points of the study

Primary outcomes	BL vs. 3rd MFU		BL vs. 6th MFU		3rd MFU vs. 6th MFU	
	Mean difference (95% CI)	*P*-value	Mean difference (95% CI)	*P*-value	Mean difference (95% CI)	*P*-value
WOMAC-pain						
QSE(*n* = 43)	1.442(from 1.129 to 1.754)	< 0.001	1.372(from 0.988 to 1.756)	< 0.001	− 0.070(from − 0.412 to 0.272)	0.946
BDJ(*n* = 41)	1.049(from 0.729 to 1.369)	< 0.001	1.293(from 0.900 to 1.686)	< 0.001	0.244(from −0.106 to 0.594)	0.257
CE(*n* = 44)	2.205(from 1.896 to 2.514)	< 0.001	3.182(from 2.803 to 3.561)	< 0.001	0.977(from 0.639 to 1.315)	< 0.001
WOMAC-physical function						
QSE(*n* = 43)	2.163(from 1.624 to 2.702)	< 0.001	2.023(from 1.065 to 2.982)	< 0.001	−0.140(from −1.025 to 0.746)	0.974
BDJ(*n* = 41)	2.439(from 1.887 to 2.991)	< 0.001	2.756(from 1.907 to 3.605)	< 0.001	0.317(from −0.394 to 1.028)	0.373
CE(*n* = 44)	4.727(from 4.194 to 5.260)	< 0.001	6.545(from 5.598 to 7.493)	< 0.001	1.818(from 0.943 to 2.694)	< 0.001

*WOMAC* Western Ontario and McMaster Universities osteoarthritis index, pain (0–20), physical function (0–68), *QSE* Quadriceps strengthening exercise, *BDJ* Baduanjin qigong, *CE* The combined exercise of quadriceps strengthening exercise and Baduanjin qigong, *BL* Baseline, *MFU* Month of follow-up, *SD* Standard deviation, *CI* Confidence interval; *p* < 0.05 was considered statistically significant after adjustment

### Effects on self-efficacy and HRQoL

Table 4 shows results for self-efficacy and HRQoL. There were no statistically significant differences among the three groups for any outcome at baseline. After 3 months of follow-up, statistically significant differences were found for self-efficacy (F = 4.123, *P* = 0.018). At 6 months of follow-up, there were statistically significant differences among the three groups in self-efficacy (F = 12.146, *P* < 0.001) and physical component summary (F = 4.233, *P* = 0.017) and mental component summary (F = 3.890, *P* = 0.023) of SF-12.

**Table 4:** Comparisons of self-efficacy and HRQoL among the three groups in different time points (*n* = 128)

Secondary Outcomes	BL	3rd MFU	6th MFU	Between groups (QSE vs. BDJ vs. CE)						Time × Group	
				BL		3rd MFU		6th MFU			
	(Mean ± SD)	(Mean ± SD)	(Mean ± SD)	*F*	*P*-value	*F*	*P*-value	*F*	*P*-value	*F*	*P*-value†
SF-12 PCS				0.062	0.940	1.741	0.180	4.233	0.017	7.470	< 0.001
QSE(*n* = 43)	41.35 ± 9.17	45.21 ± 9.23	44.33 ± 10.03								
BDJ(*n* = 41)	41.62 ± 8.40	46.88 ± 10.44	45.88 ± 11.91								
CE(*n* = 44)	42.05 ± 10.21	49.43 ± 12.04	51.42 ± 13.58								
SF-12 MCS				0.033	0.968	2.267	0.108	3.890	0.023	10.207	< 0.001
QSE(*n* = 43)	44.77 ± 11.81	48.33 ± 11.38	47.45 ± 13.97								
BDJ(*n* = 41)	45.35 ± 11.84	48.93 ± 11.28	48.40 ± 13.68								
CE(*n* = 4))	45.31 ± 11.68	52.98 ± 10.59	54.61 ± 11.25								
ASES-8				0.072	0.931	4.123	0.018	12.146	< 0.001	22.359	< 0.001
QSE(*n* = 43)	4.74 ± 0.83	5.44 ± 0.84	5.26 ± 1.17								
BDJ(*n* = 41)	4.79 ± 0.69	5.53 ± 0.81	5.72 ± 1.16								
CE(*n* = 44)	4.80 ± 0.76	5.92 ± 0.85	6.48 ± 1.22								

**†** The repeated measures ANOVA was used to test the time by group interaction effects across the 3 time points of the study

*ASES-*8 Short form-8 item arthritis self-efficacy scale, *SF-12* Short form-12 item health survey questionnaire, *SF-12 PCS* Physical component summary, *SF-12 MCS* Mental component summary, *QSE* Quadriceps strengthening exercise, *BDJ* Baduanjin qigong, *CE* The combined exercise of quadriceps strengthening exercise and baduanjin qigong, *BL* Baseline, *MFU* Month of follow-up, *SD* Standard deviation; *p* < 0.05 was considered statistically significant

The analyses of time-by-group-interaction effects on self-efficacy and HRQoL for the length of the whole study are shown in Table 4. The analysis indicated statistically significant time-by-group-interaction effects on self-efficacy (F = 22.359, *P* < 0.001), physical component summary (F = 7.470, *P* < 0.001), and mental component summary (F = 10.207, *P* < 0.001).

## Discussion

Since OA cannot be cured [1], lifestyle change, particularly ​​exercise and activity, is vital when managing knee OA [10, 11]. Therefore, it is necessary to develop effective exercise intervention programs that facilitate compliance by those with KOA.

Our study suggests that compared to an intervention only involving QSE or BDJ, a combining program of QSE and Baduanjin was more effective in reducing pain intensity in older KOA patients, improving their physical functioning, self-efficacy, and HRQoL. Crucially, the combination exercise program resulted in a better compliance (Figs. 2 and 3).

Research has shown that diminished lower limb muscle strength in KOA patients is associated with disease progression, pain, physical dysfunction, and reduced quality of life [8, 31]. Thus, quadriceps strengthening can be considered vital in managing KOA [23]. QSE as a widely-used muscle training method [7, 10, 11]. Its beneficial effects on pain relief and functional improvement in KOA patients has been demonstrated by our study (Table 2). The benefits of Baduanjin for joint pain and quality-of-life in KOA patients is similar to those of practicing QSE (Table 3). This is largely consistent with the conclusions of previous studies [32]. Pain and physical function, as measured using WOMAC, improved significantly during follow-up in the CE group when compared to the other two groups. The greater benefit of combining QSE with BDJ, in addition to the effects of QSE on leg muscle strength, may relate to how BDJ improves function, allowing greater participation in activities, in elderly KOA patients, and how this alters their pain perception.

As a low-impact aerobic exercise with soft movements, Baduanjin stretches and relaxes the musculoskeleton throughout the entire body [16]. Previous studies have suggested that Baduanjin improves fatigue, balance, and physical flexibility in older people [14, 16]. Importantly, partial Baduanjin poses [14, 33, 34] could specifically enhance lower limb muscle strength. For example, “drawing the bow on both sides” contracts hamstring muscles while stretching the quadriceps; “swaying the head and shaking the tail” exercises lower limb adductor muscles; “bouncing on the toes” simultaneously strengthens lower limb anterior and posterior muscles by putting knees together and lifting heels.

Due to the positive effects of Baduanjin movements on lower limb muscle strength, pain and physical functionality improved significantly after training in the CE group (as measured by WOMAC) (Tables 2, 3). Maintaining functionality is a main goal of OA treatment [9, 35]. Good physical function benefits patients allowing them to undertake daily activities and independent social interaction [7]. Quality of life in these patients improved correspondingly [35]. Our findings support combining QSE and Baduanjin interventions in KOA.

As a mind-body exercise, Baduanjin postures can strengthen muscles [16], regulate breathing, inducing a relaxed state of mind [18]. Thus it may help individuals decrease pain sensitivity and alter their perception of pain. Evidence demonstrates that Baduanjin training increases excitation in the middle cerebral cortex, down-regulating anxiety and negative emotions [36], and thus promotes mental health [37]. With a peaceful state of mind, individuals are more likely to make correct decisions in self-management [38], and the corresponding impact of pain may likewise be reduced [39]. Such influences help break the “pain cycle” [15], enhancing patient confidence when coping with disease or discomfort [13].

Our findings suggest that a QSE plus Baduanjin intervention has more a positive effect on self-efficacy and the QoL of KOA patients than does QSE or BDJ alone (Table 4). This is to be expected, as previous studies have also indicated positive associations between self-efficacy and pain in those suffering with chronic musculoskeletal pain [40]. Veenhof (2006) identifies that to ensure good compliance in OA patients, the ultimate goal of exercise intervention is to integrate the exercises into daily life [41]. However, the optimal way to achieve this remains an open question. The reality is that this goal is seldom achieved by asking patients to perform monotonous exercises repetitively [12]. Instead, it is essential to mobilize multifaceted support around the patient, such as encouragement from peers [42–44].

In our study, participants were required to practice Baduanjin using a group-based form, so that they could share successful personal experience in OA management [44] and to provide mutual encouragement and support [42]; participants in the QSE group practiced QSE at home alone. Moreover, group exercise may make patients feel exercise is not a treatment but a part of daily life, eliminating their awareness of a patients’ role and enhancing their sense initiative in KOA management. Thus, subjects in the CE group had higher levels of self-efficacy and exhibited better exercise adherence.

A review indicated that choosing exercises based on patient preference can increase adherence to exercise [12]. Baduanjin is very popular among older adults, but most such patients with KOA were concerned that practicing Baduanjin might aggravate joint symptoms [45]. In our study, such concerns were deprecated through appropriate guidance by researchers. Our study indicates that practicing Baduanjin was safe and effective for older patients with KOA, and that participants in the QSE plus Baduanjin group demonstrated greater adherence. Adherence to exercise was a key predictor of beneficial long-term outcome from exercise [8]. When designing the study, our concern was that the greater amount of exercise expected of the intervention group might reduce compliance. However, at 6 months of follow-up, results indicated that the CE group exhibited greater enthusiasm, participation, and adherence.

There were several limitations in our study. First, participants were only recruited from urban communities, while rural residents were not included. Ideally, future investigation should include rural residents from diverse settings, so that a more detailed and conclusive assessment of the effectiveness of the program can be obtained. Secondly, patients who agreed to participate in the study might be more concerned about their health, resulting in higher completion and lower patient attrition rates, and ultimately achieving better intervention results. Thirdly, to maintain safety, we only recruited older patients with mild to moderate pain on NRS. Future study should include patients with severe pain to confirm the efficacy of QSE plus Baduanjin treatment in reducing pain and improving other health outcomes among such patients. Finally, a longer period of follow-up should be used to test long-term compliance and outcomes. Larger, more strictly designed trials are needed to confirm our findings.

## Conclusions

To conclude, exercise programs combining strengthening exercise and Baduanjin promote positive lifestyle changes with increased physical activity. Results indicate that patients with KOA in the QSE plus Baduanjin group exhibited significantly greater pain reduction, greater improvement in physical function and self-efficacy, and a better physical and mental health status than those in the QSE or BDJ only group. Likewise, outcome improvements in the QSE plus Baduanjin group are better sustained. When practiced correctly, Baduanjin exercise also appears to be safe and effective for patients with KOA. This study provides evidence of the value of exercise programs for patients with KOA. Due to the small sample size of our study, our conclusion should be treated with caution, indicating the need for longer, larger, and more sophisticated studies to evaluate the efficacy of combining Baduanjin exercise with QSE.

## Supplementary Information

**Additional file 1.** The guidelines for practicing quadriceps strengthening exercises and Baduanjin qigong for KOA patients in this study.

**Additional file 2.** The dataset supporting the conclusions of this article.

**Additional file 3.** CONSORT 2010 checklist of information to include when reporting a randomized trial.

## Data Availability

The dataset supporting the conclusions of this article can be accessed through Additional file 2.

## Abbreviations

OA: Osteoarthritis
KOA: Knee osteoarthritis
NRS: Numeric rating scale
BMI: Body mass index
WOMAC: Western Ontario and McMaster Universities Osteoarthritis Index
ASES-8: Short Form-8 item Arthritis Self-Efficacy Scale
SF-12: Short Form-12 item health survey questionnaire
SF-12 PCS: Physical component summary of SF-12
SF-12 MCS: Mental component summary of SF-12
QSE: Quadriceps strengthening exercises
BDJ: Baduanjin qigong.
CE: The combined exercise of quadriceps strengthening exercise and baduanjin qigong
HRQoL: Health related quality of life
BL: Baseline
MFU: Month of follow-up
CI: Confidence interval
SD: Standard deviation
