# Supplementary material for: The effects on pain, physical function, and quality of life of quadriceps strengthening exercises combined with Baduanjin qigong in older adults with knee osteoarthritis: a quasi-experimental study
Source: BMC Musculoskelet Disord. 2021 Mar 29;22:313. doi: 10.1186/s12891-021-04179-8 (PMC8008642; doi:10.1186/s12891-021-04179-8)
Supplement: Supplementary file 1 — Additional file 1. The guidelines for practicing quadriceps strengthening exercises and Baduanjin qigong for KOA patients in this study. [file 12891_2021_4179_MOESM1_ESM.docx]

**Additional file 1 :** **The guidelines for practicing quadriceps strengthening exercises and Baduanjin qigong for KOA patients in this study**

***1. Quadriceps strengthening exercises (QSE) includes the following four items.***

(1) Straight leg raising: The participant, lay in supine position with one leg flexed to about 45° to support the bed surface, while contracting quadriceps of the other leg and lifting the lower extremity up to approximately 20 cm away from the bed while maintaining the knee in extension. This position was held for 5–10 seconds, then the limb was lowered slowly. This was repeated 10 times.

(2) Isometric contractions of the quadriceps: The participant lay in supine position while contracting the quadriceps muscle of the affected limb with maximum strength and maintaining the knee in extension. The contraction was held for 5–10 seconds, then relaxed. This was repeated 10 times.

(3) Leg flexed and extended in the sitting position: The participant sat on a chair keeping the upper body straight while raising the affected limb and extending the knee with ankle dorsiflexion. This position was held for 5–10 seconds, then the limb was retracted slowly. This was repeated 10 times.

(4) Mini-squats: While standing, the participant bent both knees by about 30–60°, while maintaining the trunk upright. This position was maintained for 5–10 seconds, relaxed, and repeated 10 times.

***2. Baduanjin qigong*** recommended by Chinese Health Qigong Association in 2003, consisted of eight postures (the materials can be accessed online: https://v.youku.com/v_show/id_XNDEzOTI3NzU4OA==.html) .


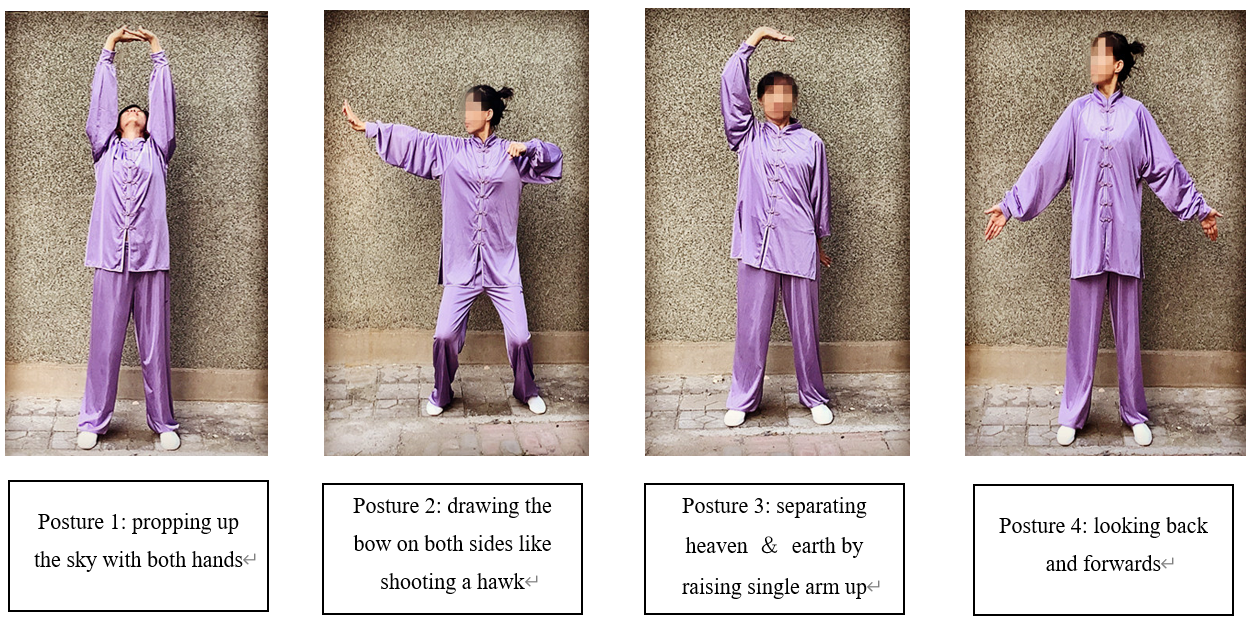


***
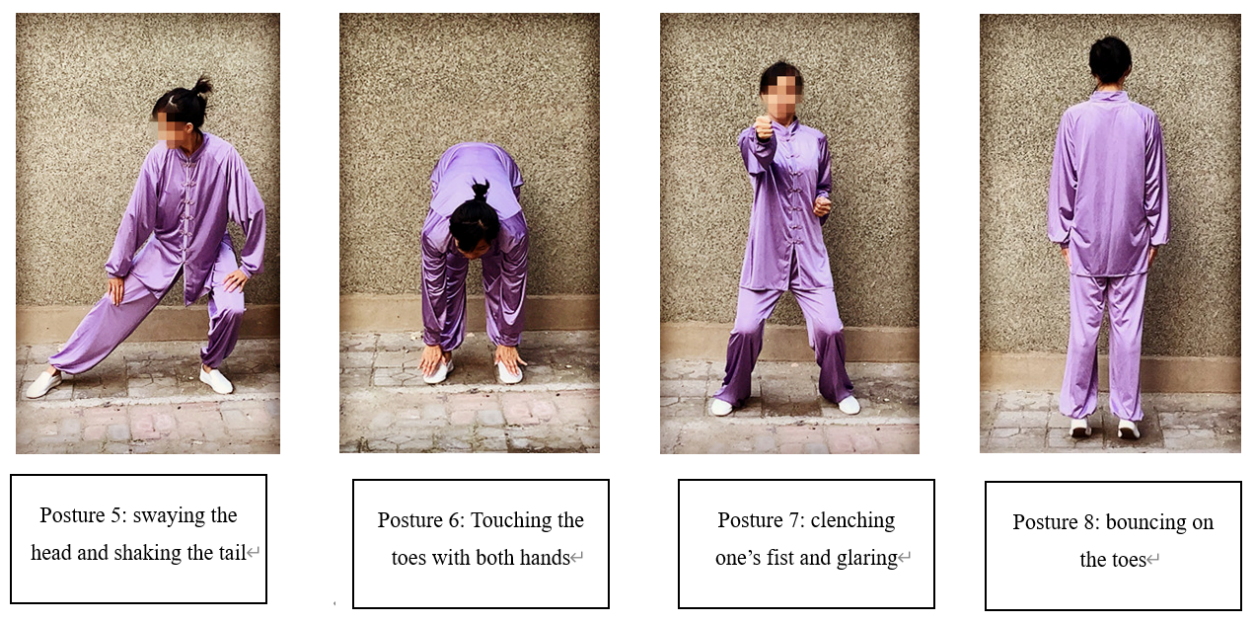
***

Fig. 1. Illustration of the 8 postures of Baduanjin qigong

**The movements of Baduanjin qigong are detailed as follows:**

**Preparation posture:**

(1) Step left and stand with feet shoulder width apart.

(2) Squat with keens bending slightly.

(3) Lower arms adducted with palms facing to the belly.

**Posture 1: propping up the sky with both hands**

**(1) Crosse the hands in front of the belly.**

(2) Raise the hands slowly with palms facing upward.

(3) Turn the palms out at the chest level.

(4) Continue to raise the palms overhead until the elbows stretch straight.

(5) Look upward and lift your heels as extending the arms (if possible).

(6) Put down the arms sideways with the palms facing down and heels to the ground.

(7) Return to the starting stance.

(8) Repeat steps (1) - (7) for 6 times.

**Regulate respiration:** inhale as raising the hands and exhale as lowering them.

**Posture 2: drawing the bow on both sides like shooting a hawk**

(1) Step left to a wide horse stance.

(2) Cross the forearms in front the chest with the right arm in front of the left.

(3) Stretch out the left arm with the index and the middle fingers erect and other fingers bent. At the same time, bend the right arm while making a fist and draw the elbow to the right side. Meanwhile, bend the knees slightly as if riding on a horse and drawing a bow.

(4) Release the right hand and let the arrow fly.

(5) Drop the arms with palms down as the body floats up.

(6) Back to preparation posture and start the other side.

(7) Repeat steps (2) – (6) on the right side.

(8) Repeat the movements on both the left and right sides for 3 times.

**Regulate respiration:** Inhale as extending the arm out (drawing a bow) and exhale as releasing the arm (returning to the starting pose).

**Posture 3: separating heaven ＆ earth by raising single arm up**

(1) Start in a preparation posture with a small step sideways.

(2) Lift the hands to the stomach with palms facing upward.

(3) Continue the movement by raising the left hand until the left arm is extended over your head with the palm up and fingers points to the right.

(4) At the same time, pressing the right hand down with the palm down and fingers points to the front.

(5) Return to the starting pose by lowering the left hand in front of your body.

(6) Repeat steps (2) - (5) with the right arm up and the left arm down.

(7) Repeat the movements on both sides for 3 times.

**Regulate respiration:** Inhale as lifting the arm and exhale as lowering the arm.

**Posture 4: looking back and forwards**

(1) Start in a preparation posture with a small step sideways.

(2) Straighten legs and extended the arms down at the body sides with palms out

(3) At the same time, turn the head slowly to the left and the eyes gaze behind as far as you can. Keep the body up straight while looking back.

(4) Back to the starting pose with the head return to the center and the arms rotating inward.

(5) Repeat steps (2) - (4) on the right side.

(6) Repeat the movements on both sides for 3 times.

**Regulate respiration:** Inhale as turning the head backward and exhale as returning the head to the center.

**Posture 5: swaying the head and shaking the tail**

(1) Step left to a wide horse stance.

(2) Put the hands up over head and straighten legs.

(3) Drop the arms slowly, bend the knees and squat down lower. Keep the back straight and the knees are centered over the toes.

**(4) Place hands just above the knees with the thumbs on the outside of the legs and the elbows pointed to the sides.**

**(5) Shift gravity center to the left, turn the chest to the left, and the tailbone to the right. Extend the right leg to help the body turn to the left.**

**(6) Move the upper body from the left to the right and the gravity center shifts to the right foot with straightening the left leg. keep the back straight.**

**(7) Sway the head when the body turn until the rightmost, then shift back to**

center.

(8) Repeat steps (5) - (7) with shifting the gravity center from the right to the left.

(9) Repeat the movements on both sides for 3 times.

**Regulate respiration:** Inhale as lean down, exhale as the head **sway**ing ahead, inhale as the head **sway**ing back, exhale as returning to center.

**Posture 6: Touching the toes with both hands**

(1) Start in a preparation posture.

(2) Straighten legs and extended the arms up **over the head.**

**(3) Bend the arms down at the elbows with fingertips facing each other.**

**(4) Rotate the wrist to palms upside and then the palms slide from the chest to the back.**

**(5) Put palms on the back at the kidneys.**

**(6) Lean down slowly with the hands sliding down from the spine to the instep.**

**(7) Stretch palms forward by the ear side, drive the upper body straight by arms.**

(8) Repeat steps (2) - (7) for 6 times.

**Regulate respiration: inhale as arms up, exhale as arms down; inhale as rotate wrist, exhale as lean down; inhale as body up, exhale as body down.**

**Posture 7: clenching one’s fist and glaring**

(1) Step left to a wide horse stance.

(2) Drop the torso, clench a tight fist with both hands beside the waist with palms up.

(3) punch out forward with the left hand.

(4) Spread palm and rotate the left wrist one hundred and eighty degree counterclockwise. And then fingers curled tightly into a fist, end with the left fist palm down. Clench the teeth tightly and open eyes wide as if you were angry. Toes should grip the ground firmly.

(5) Bring the left fist back to the starting pose.

(6) Repeat steps (2) - (5) on the right side.

(7) Repeat the movements on both sides for 3 times.

**Regulate respiration:** inhale as withdrawing the fist and exhale as punching.

**Posture 8: bouncing on the toes**

(1) Stand at ease and keep the back straightened.

(2) Lift the heels and raise the body.

(3) Lower the heels suddenly to give the whole body a little shock. For those who are not very strong, lower the heels gently instead of suddenly.

(4) Repeat steps (2) - (3) for 7 times.

**Regulate respiration:** inhale as lifting the heels and exhale as lowering them.

**Closing posture:**

(1) Stand upright with feet shoulder width apart.

(2) Crosse the palms and place them on the Dantian point, with the thumbs at navel level.

(3) Relax the whole body and feel the Qi sinking into Dantian.
